# Aortoenteric fistula following overlap esophagojejunal anastomosis using linear staplers for cancer of the esophagogastric junction: a case report

**DOI:** 10.1186/s40792-019-0566-0

**Published:** 2019-01-16

**Authors:** Masayuki Honda, Tsuguo Sakamoto, Shigehiro Kojima, Yota Yamamoto, Kazuhito Yajima, Dal Ho Kim, Fumihiro Ogawa

**Affiliations:** Department of Surgery, Sainokuni Higashiomiya Medical Center, 1522, Torocho, Kitaku, Saitama, Saitama 331-8577 Japan

**Keywords:** Aortoenteric fistula, Esophagogastric junction, Linear stapler, Overlap anastomosis

## Abstract

**Background:**

Aortoenteric fistula (AEF), occasionally reported as a fatal complication after aortic or other vascular procedures, is a communication between the aorta and the digestive tract. AEF as a fatal complication of overlap esophagojejunostomy after esophagogastrectomy has not been reported previously. Herein, we report a case of AEF after laparoscopic proximal gastrectomy and transhiatal lower esophagectomy for cancer of the esophagogastric junction, in which linear staplers were used for overlap esophagojejunostomy.

**Case presentation:**

A 66-year-old woman with advanced cancer of the esophagogastric junction underwent laparoscopic proximal gastrectomy and transhiatal lower esophagectomy with abdominal and lower mediastinal lymphadenectomy. Double tract reconstruction by the overlap method was performed. The patient was discharged from the hospital 10 days after surgery with a good postoperative course. However, she developed sudden-onset massive hematemesis and melena the day after discharge, resulting in death. Autopsy revealed that the stapled edge of the entry hole of the overlap esophagojejunostomy was in direct contact with the descending aorta. AEF was found at the esophagojejunostomy site.

**Conclusions:**

To our knowledge, this is the first report of AEF as a fatal complication of overlap esophagojejunostomy after esophagogastrectomy. Although we could not definitively identify the cause of the AEF, it could be attributed to direct contact between the stapled edge and the bare thoracic aorta over a period of 10 days. To avoid direct contact with the aorta in esophagojejunostomy with linear staplers, all stapled edges should be covered by suturing and attention should be paid to the position of these edges.

## Background

Esophagojejunostomy using linear staplers (overlap method) after laparoscopic total gastrectomy for gastric cancer was first reported by Inaba et al. [1]. To date, several reports have stated that overlap esophagojejunostomy is a safe and useful method after laparoscopic total gastrectomy [2–4]. One rare report has detailed good postoperative outcomes of seven patients after esophagogastrectomy with radical lymphadenectomy in the lower mediastinum followed by overlap esophagojejunostomy [5]. Aortoenteric fistula (AEF) at the site of overlap esophagojejunostomy after laparoscopic total gastrectomy has been reported in the literature [6]. Herein, we report a case of AEF at the esophagojejunostomy site after laparoscopic esophagogastrectomy for cancer of the esophagogastric junction (EGJ), which is a rare, fatal complication involving the development of a fistula between the aorta and digestive tract. The present case report demonstrates the possible association between the overlap esophagojejunostomy with linear staplers and AEF.

## Case presentation

A 66-year-old Japanese woman was referred to our hospital with complaints of vomiting. Endoscopic upper gastrointestinal imaging revealed a type 3 tumor at the EGJ (circumference, 56 mm) with stenosis. The epicenter was 3 mm from the EGJ on the gastric side. Computed tomography (CT) showed lymph node metastases along the lesser curvature of the stomach and the proximal splenic artery. The patient was diagnosed with a cT4aN2M0, cStage IIIC lesion according to the Union for International Cancer Control’s TNM classification [7].

## Operative findings

**Fig. 1:**
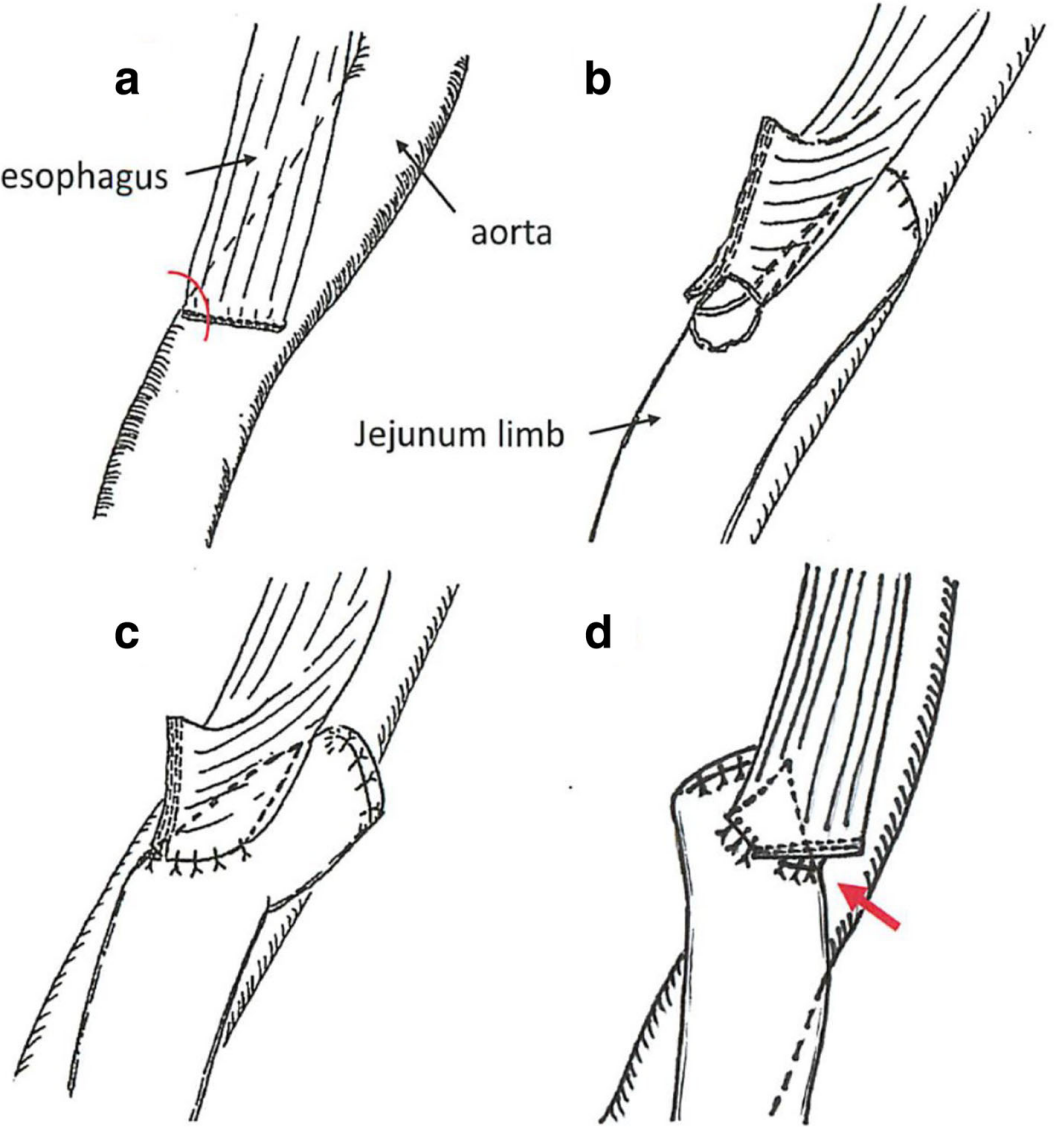
Schematic diagram of overlap esophagojejunostomy. **a** Latitudinal dissection of the esophagus via a left 8th intercostal trocar with linear stapler to obtain a safety margin. The right side of the stapled edge was cut. **b** Creation of side-to-side anastomosis created with the linear stapler between the right side of the esophagus edge and the jejunal limb, after which the jejunal staples were covered by seromuscular sutures extracorporeally. **c** The communication hole was closed using an interrupted hand-sewn technique. **d** The dorsal edge of the stapler was in contact with the bare thoracic aorta (arrow)

Laparoscopic proximal gastrectomy with abdominal lymphadenectomy was performed. After the diaphragm was separated from the hiatus using a linear stapler, radical lymph node dissection in the lower mediastinum was performed. The esophagus was latitudinally dissected with a linear stapler via a left 8th intercostal trocar. Double tract reconstruction by intrathoracic overlap method was performed using linear staplers (Fig. 1). A side-to-side anastomosis was created between the right side of the esophagus and jejunal limb, and the jejunal staples were extracorporeally covered by seromuscular sutures. Finally, the entry hole was closed intracorporeally using the interrupted hand-sewn technique [4, 8].

## Histopathological findings

The tumor was histopathologically diagnosed as a moderately to poorly differentiated squamous cell carcinoma of the EGJ. Lymph node metastases were found in 22 of 50 removed nodes, and the pathological stage was pT3N3aM0, stage IIIB according to the Japanese Gastric Cancer Association Classification 14th. R0 resection was achieved.

## Postoperative course

**Fig. 2:**
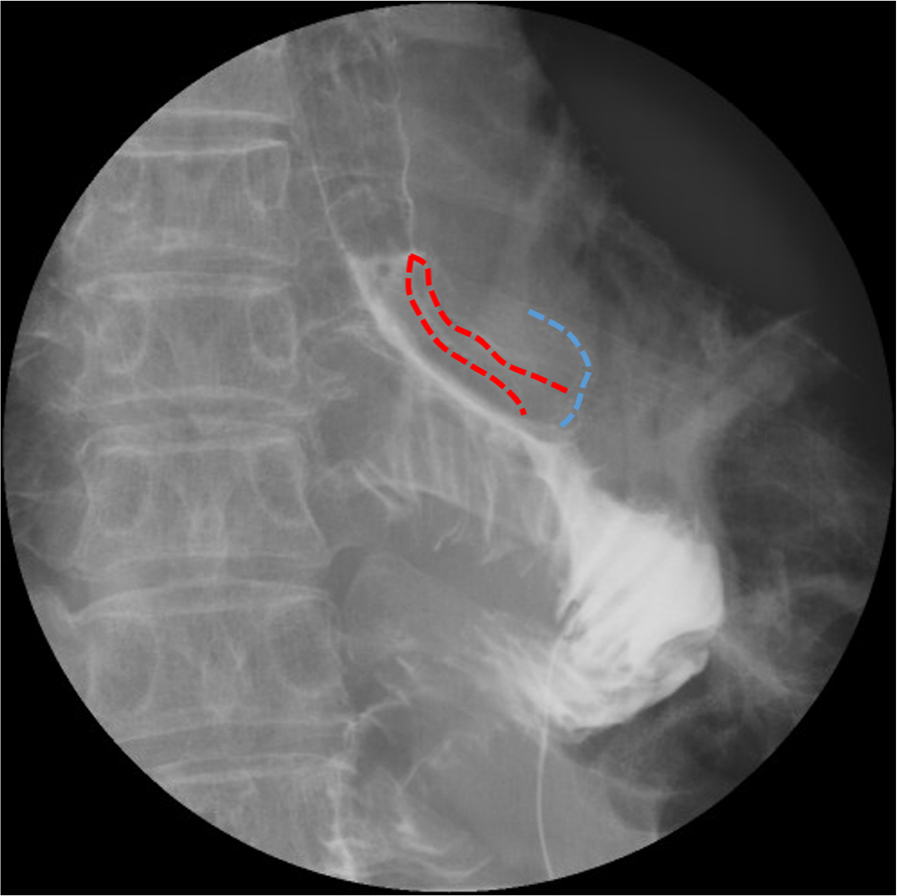
X-ray image with contrast medium on postoperative day 2 revealing no leakage of the esophagojejunostomy. Esophageal stump (dashed blue line) and stapled lines of the entry hall (dashed red line) can be seen

**Fig. 3:**
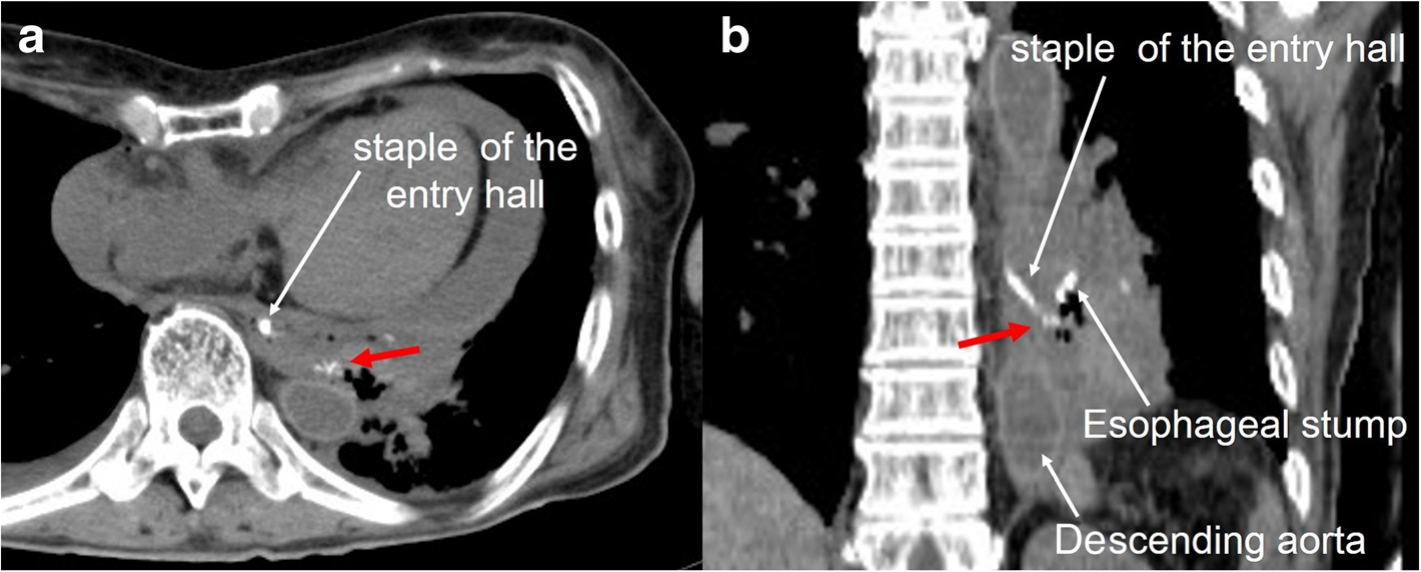
Post-mortem computed tomography showing that the dorsal stapled edge (red arrow) of the entry hole was in direct contact with the bare descending aorta. **a** Axial plane. **b** Coronal plane

**Fig. 4:**
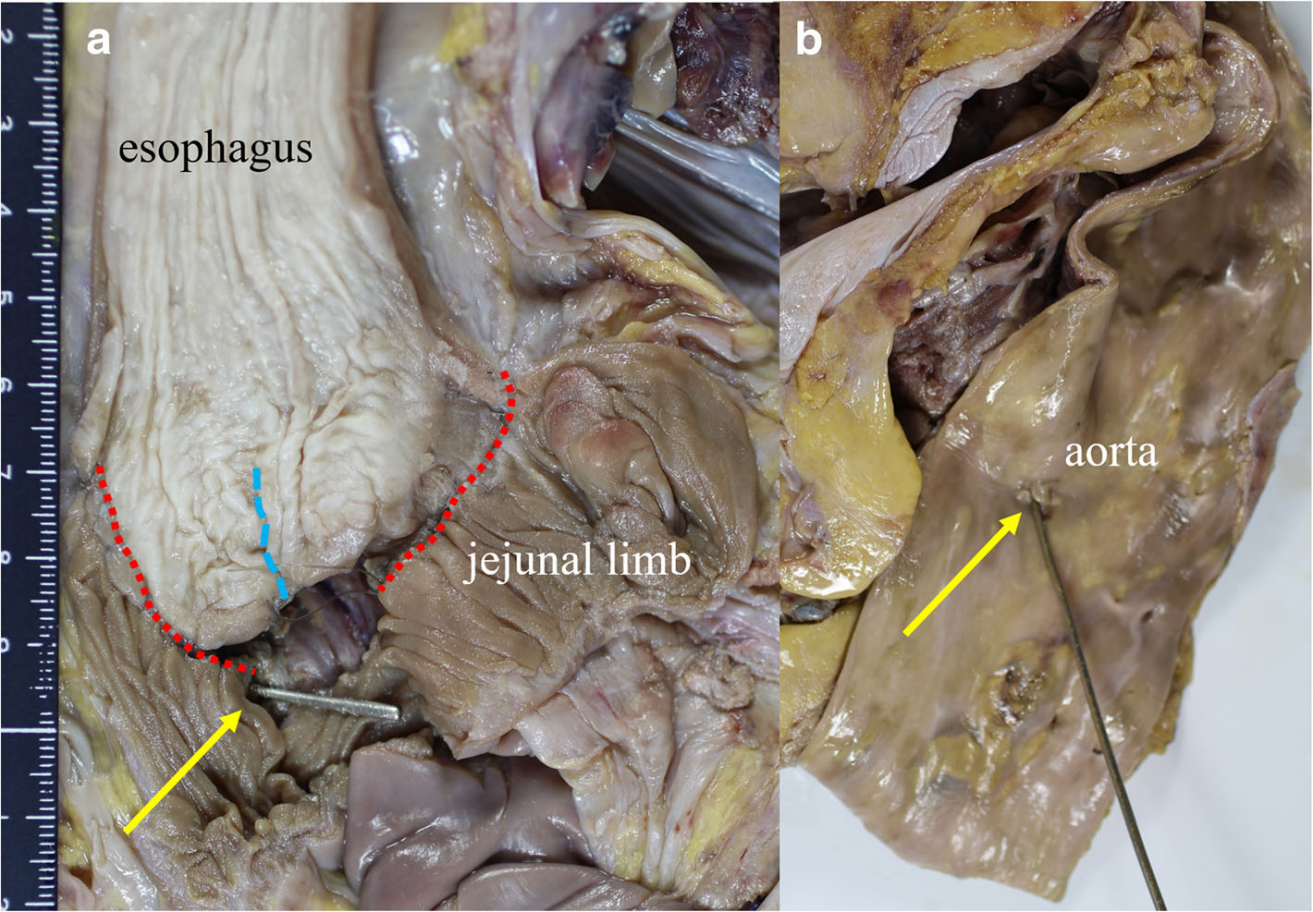
Autopsy revealed a fistula at the site of the anastomosis, located at the point between the stapled edge of the entry hall and the aorta (arrow). The dashed red line shows the staples of the entry hall, and the blue line shows the staples at the esophageal stump. **a** Esophagojejunostomy side. **b** Aortic side

**Fig. 5:**
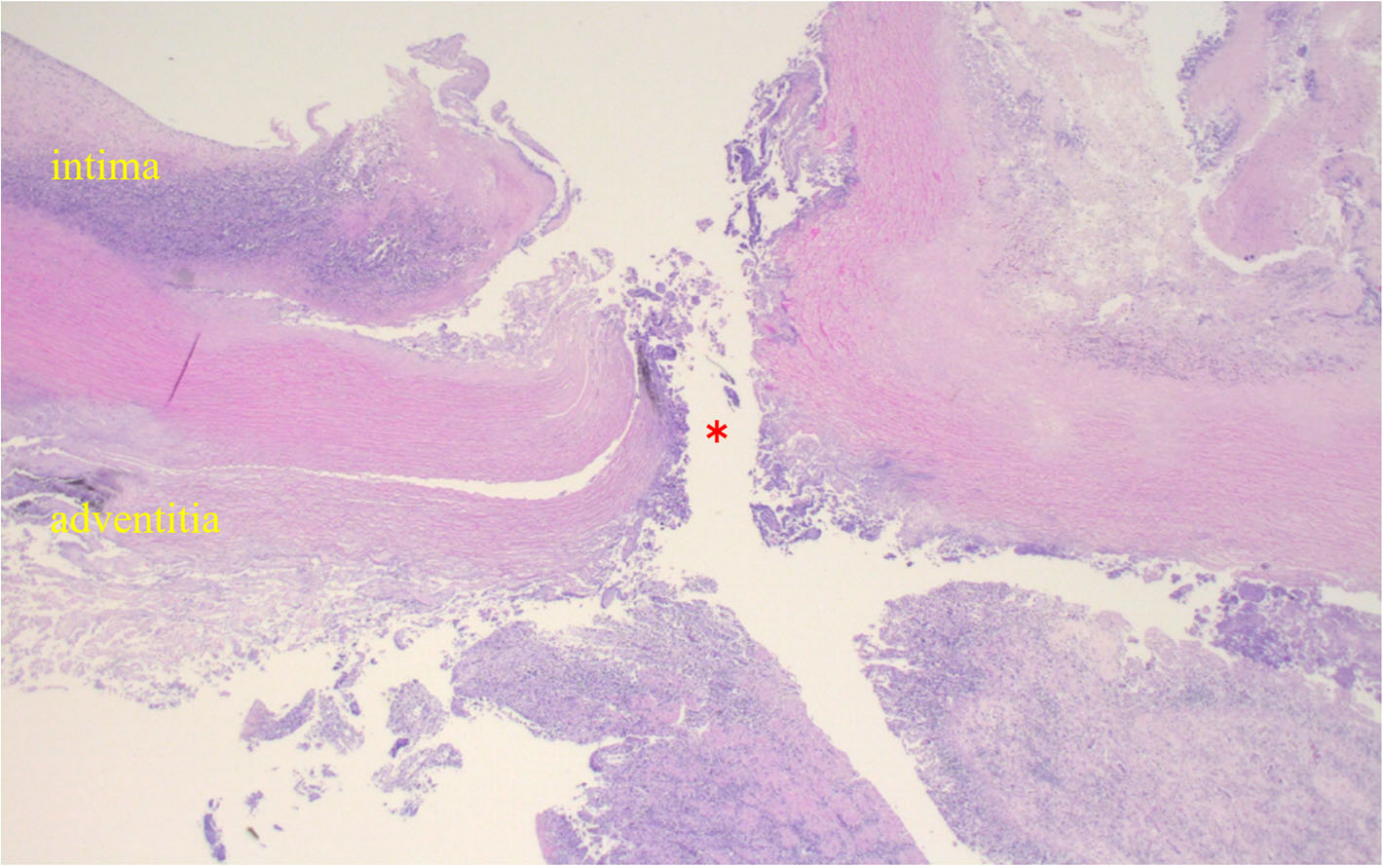
Microscopic findings showing a fistula between the aortic wall and the overlap esophagojejunostomy site. The fistula appears as a tract from the adventitia to the intima of the aorta (asterisk). There is no thermal denaturation of the adventitia of the aortic wall, and only a small amount of neutrophils are present in the intima

On postoperative day (POD) 2, upper gastrointestinal series revealed no leakage of the esophagojejunostomy (Fig. 2). The patient resumed normal diet on POD 3 and was discharged on POD 10. However, on the next day, she developed melena followed by massive hematemesis and was transferred to our hospital in cardiopulmonary arrest. Post-mortem CT revealed that the dorsal stapled edge of the entry hole was in direct contact with the bare descending aorta (Fig. 3). An AEF at the esophagojejunostomy site was revealed on autopsy (Fig. 4). Neither hemorrhage nor major abscess was observed in the thoracic and abdominal cavities. Microscopic findings demonstrated a fine tract from the adventitia to the intima of the descending aorta. There were only a few neutrophils in the intima of the aortic wall (Fig. 5). These findings indicated that the fistula was created by direct contact of the stapled edge and not by any adjacent abscess due to leakage of the anastomosis. The cause of death was considered to be hemorrhagic shock due to AEF.

## Discussion

AEFs are abnormal communications between the aorta and bowel and are commonly caused by thoracic aortic aneurysms, esophageal foreign bodies, esophageal cancers, and post-vascular surgical complications [9, 10]. Open total gastrectomy is commonly associated with anastomotic leakage, pancreatic fistula, abdominal abscess, intestinal obstruction, and pneumonia, with AEF being a rare complication [11–13]. Overlap esophagojejunostomy has been reported to be a safe and useful technique in laparoscopic total gastrectomy [1–3, 8]. Gunji et al. reported a case of AEF after laparoscopic total gastrectomy with overlap esophagojejunostomy for advanced gastric cancer with esophageal invasion [6]. In the reported case, bacterial peritonitis was diagnosed 5 days after the operation, manifesting as partial necrosis and perforation of the small intestine. The patient was treated successfully with laparoscopic partial resection of the small intestine, but unfortunately died of massive hematemesis due to AEF 30 days after her initial procedure. The authors hypothesized that AEF could have resulted from persistent contact and subsequent abrasion by the staple line on the aortic wall.

In our case, autopsy revealed the dorsal stapled edge of the entry hole located on the surface of the descending aorta (Fig. 4). The microscopic findings demonstrated a fine tract from the adventitia to the intima of the aorta, where the EGJ staple was stuck. The stapled edge was speculated to be exposed outside the anastomosis owing to the interrupted hand-sewn technique without inverting the stapled edges inside. The causes of the AEF were considered to be the sustained contact between the exposed staple and aorta and the power of each heartbeat. The position of the jejunal limb was on the right side of the esophagus, and the dorsal staple was in contact with the aorta owing to inadequate mobilization of the thoracic esophagus (Fig. 1d).

Noshiro stated that some extraverted esophageal and intestinal stumps in the thorax should be covered by suturing to prevent the formation of pulmonary fistulas, although AEF was not mentioned [5]. Gunji et al. reported an AEF located above a triangular anastomosis between the esophagus, jejunal limb, and the descending aorta [6]. They speculated that the persistent contact of the staple line eroded the aortic wall, leading to AEF. We suggest that all staples should be covered with suturing to prevent contact with the aorta. A side-to-side anastomosis could be created between the dorsal side of the esophagus and the jejunal limb, forming a cushion between the staples and the aorta.

## Conclusions

In this report, we describe a rare case of AEF, a lethal complication post-esophagojejunostomy with linear stapler after laparoscopic esophagogastrectomy. Although we could not definitively identify the cause of the AEF, it could be attributed to the direct contact between the stapled edge and the bare thoracic aorta over a period of 10 days. To avoid direct contact with the bare aorta during anastomosis with linear staplers, all stapled edges should be covered by suturing, and attention should be paid to the position of the jejunal limb.

## Abbreviations

AEF: Aortoenteric fistula
EGJ: Esophagogastric junction
POD: Postoperative day
